# Zeitmanagement im OP – eine Querschnittstudie zur Bewertung der subjektiven und objektiven Dauer chirurgischer Prozeduren im HNO-Bereich

**DOI:** 10.1007/s00106-021-01119-9

**Published:** 2021-11-14

**Authors:** Lena Zaubitzer, Annette Affolter, Sylvia Büttner, Sonja Ludwig, Nicole Rotter, Claudia Scherl, Sonia von Wihl, Christel Weiß, Anne Lammert

**Affiliations:** 1 Klinik für Hals-Nasen-Ohren-Heilkunde, Kopf- und Halschirurgie, Universitätsklinikum Mannheim, Medizinische Fakultät Mannheim, Universität Heidelberg, Theodor-Kutzer-Ufer 1–3, 68167 Mannheim, Deutschland; 2 Medizinische Statistik, Biomathematik und Informationsverarbeitung, Medizinische Fakultät Mannheim, Universität Heidelberg, Mannheim, Deutschland

**Keywords:** Chirurg, Ausbildung, Schnitt-Naht-Zeit, Planung, Wirtschaftlichkeit, Einschätzung, Surgeon, Training, Cut-suture time, Planning, Economy, Assessment

## Abstract

**Hintergrund:**

Die Gestaltung des Operations(Op.)-Programms im klinischen Alltag ist von hoher Wichtigkeit für die Wirtschaftlichkeit. Gleichzeitig muss die Einhaltung von Arbeitszeiten unterschiedlicher Berufsgruppen berücksichtigt werden.

**Ziel der Arbeit:**

Um Fehlerquellen bei der Planung aufzudecken, wurden durch Chirurgen geschätzte mit objektiv erhobenen Zeiten (u. a. Schnitt-Naht-Zeit) verglichen.

**Material und Methoden:**

In einer retrospektiven Analyse wurden 1809 Operationen im Jahr 2018 (22 verschiedene Op.-Arten) durch 31 Operateure (12 Fach- [FÄ] und 19 Assistenzärzte [AÄ]) hinsichtlich ihrer Dauer verglichen und mittels Mann-Whitney-U-Test auf Signifikanz geprüft.

**Ergebnisse:**

Der Vergleich der objektiven Zeiten von FÄ und AÄ zeigt signifikante Unterschiede in der Schnitt-Naht-Zeit bzw. der Summe aus Schnitt-Naht-Zeit und Zeit der chirurgischen Maßnahmen für 6 von 15 Op.-Arten (*p* < 0,001). Die durch FÄ geschätzte Nachbereitungszeit wich bei 2 von 22 Op.-Arten von der objektiven Zeit ab (*p* < 0,05), die durch AÄ geschätzte Zeit bei 7 von 15 Op.-Arten (*p* < 0,05). Hinsichtlich der Schnitt-Naht-Zeit verschätzten sich FÄ bei 7 von 22 (*p* < 0,05), AÄ bei 3 von 15 (*p* < 0,05) Op.-Arten. Die durch FÄ geschätzte Vorbereitungszeit wich bei 16 von 22 Op.-Arten signifikant von der objektiven Zeit ab (*p* < 0,05), bei AÄ bei 7 von 15 (*p* < 0,001). Vor- und Nachbereitungszeiten wurden durch FÄ unter‑, Schnitt-Naht-Zeiten überschätzt. AÄ unterschätzten alle Zeiten.

**Schlussfolgerung:**

Bei der OP-Planung muss die Erfahrung des durchführenden Chirurgen berücksichtigt werden. Eine Verbesserung durch verminderte subjektive Fehleinschätzung kann möglicherweise mithilfe spezieller Algorithmen gelingen.

## Abstract (en)

**Background:**

Accurate planning of operating times in surgical clinics is essential. Moreover, high-capacity utilization of operating rooms (ORs) is necessary for economic efficiency.

**Objective:**

Most planning of operating times is performed by surgeons. Herein, surgeons’ estimated times and the objective times for performing surgical procedures were compared to detect sources of error.

**Materials and methods:**

In a retrospective analysis, the durations of 1809 operations using general anesthesia (22 types of surgery) by 31 surgeons (12 specialists and 19 residents) were compared. Comparisons were analyzed by Mann–Whitney U‑tests.

**Results:**

The comparison of objective times of surgical action showed significant differences between specialists and residents in 6 of 15 types of surgeries. The post-processing times estimated by specialists deviated from the objective times in 2 out of 22 surgery types, while the post-processing times estimated by residents deviated in 7 of 15 types. Specialists misjudged the incision-to-suture times in 7 of 22 surgery types, and residents misjudged these times in 3 of 15 types. The preparation times estimated by specialists deviated from the objective times in 16 of 22 types of surgeries and in 7 of 15 types estimated by residents.

**Conclusion:**

A surgeon’s routine must be carefully considered in order to estimate operating times. Specialists generally underestimated preparation and post-processing times and overestimated incision-to-suture times, whereas residents underestimated all three. Preparation and post-processing times must be considered in planning and, ideally, determined together with anesthesiologists and surgical assistants.

## Hintergrund

In den Jahren 1995 bis 2006 haben sich die Gesundheitsausgaben um 2,5 % pro Jahr erhöht, und Krankenhäuser stellen dabei die kostenintensivsten Einrichtungen dar. Der Operationssaal (OP) ist ein wesentlicher Bestandteil der chirurgischen Versorgung unserer Patienten und eine der wichtigsten Einnahmequellen. Zusammen mit den Intensivstationen ist er aber einer der kostenintensivsten Bestandteile eines Klinikums [4, 21]. Im Jahr 2010 wurden die Kosten einer Operations(Op.)-Minute in Deutschland mit 10 bis 120 € veranschlagt [5]. Die drei wichtigsten Ressourcen im OP-Management sind dabei der OP selbst, Zeit und das spezialisierte Personal, deren Nutzung optimiert werden muss, um maximalen Gewinn zu erreichen und Kosten zu minimieren [1, 10]. Einer der wichtigsten Bestandteile eines effizienten OP-Managements ist daher die optimale Versorgung von Patienten unter einer angemessenen, effizienten Planung der Op.-Falldauer. Überschätzungen der Falldauer führen möglicherweise zu Leerlaufzeiten. Aber auch das Unterschätzen von Falldauern kann zur Folge haben, dass anschließend geplante Prozeduren ausfallen müssen, wenn es zu „Überplanungen“ kommt. Das enorme Aufkommen von Überstunden bei ärztlichem und pflegendem Personal und von Wartezeiten bei Patienten kann zu Personalfluktuation und Patientenunzufriedenheit führen [10]. Zusätzlich können chirurgische Eingriffe unvorhergesehen mehr Zeit benötigen, wenn unerwartete Komplikationen oder Befunde auftreten, die zu Anpassungen des chirurgischen Eingriffs führen und somit zusätzliche Zeit erfordern. Andererseits gibt es mehrere Faktoren, die die voraussichtliche Dauer einer Operation verkürzen können. Da die genaue Schätzung der Operationszeiten eine Voraussetzung für eine effiziente OP-Planung ist, ist es wichtig festzustellen, ob die von Chirurgen geschätzten OP-Zeiten mit den „realen“ OP-Zeiten vergleichbar sind.

Bei der Operationsplanung ergeben sich somit potenzielle Fehlerquellen. Häufig wird die Operationsplanung durch die durchführende chirurgische Abteilung gemacht und dabei spielt die subjektive Einschätzung von Falldauern durch die planende Abteilung eine wichtige Rolle. Steigender wirtschaftlicher Druck führt dazu, dass die planenden Verantwortlichen die teure OP-Kapazität keinesfalls ungenutzt lassen, um Leerlaufzeiten zu vermeiden, ohne die Sicherheit und Qualität der Patientenversorgung zu gefährden. Möglicherweise besteht daher eine Tendenz zur Unterschätzung von Falldauern, um Leerlaufzeiten zu vermeiden. Es ergibt sich die Frage, inwiefern die subjektive Einschätzung von Falldauern im OP mit objektiv erhobenen Daten übereinstimmt. Wright et al. versuchten, in ausgewählten Fällen die Zeitschätzungen von Chirurgen mit denen kommerzieller Planungssoftware zu vergleichen, um zu beurteilen, ob durch Regressionsmodellierung Verbesserungen erzielt werden könnten. Interessanterweise übertrafen die Chirurgen im Allgemeinen die kommerzielle Planungssoftware bei der Planung von OP-Zeiten. Einzelne Chirurgen waren im Vergleich zur Software sogar noch besser. Ein einfaches Modell, das die Schätzungen der Chirurgen mit historischen Daten kombiniert, reduzierte die Vorhersagefehler erheblich. Sie stellten auch fest, dass Chirurgen einen Anreiz brauchten, um ihre Fehler bei der Schätzung der Dauer zu reduzieren. Welches System auch immer zur Planung von OP-Zeiten verwendet wird, genauere Schätzungen der Dauer jedes Falls und der erforderlichen Ressourcen sollten dazu beitragen, die Unterauslastung von Ressourcen und die Überplanung zu reduzieren [11, 15, 22].

Zusätzlich ergibt sich gerade an ausbildenden Kliniken die Situation, dass Operationen deutlich länger dauern, wenn es sich um Lehreingriffe handelt [6]. Aufgrund ihrer geringeren Routine und praktischen Erfahrung benötigen Assistenzärzt*innen (AÄ) in der Regel mehr Zeit für Operationen. Daher sollte die Erfahrung des Operateurs bei der OP-Planung immer respektiert werden. Frühere Studien haben ergeben, dass die Durchführung von Lehroperationen nicht nur zu längeren Operationsdauern, sondern auch zu steigenden Kosten führt. Pollei et al. konnten zeigen, dass beispielsweise eine Tonsillektomie 11 min, eine Tympanomastoidektomie sogar 51 min länger dauert, wenn die Eingriffe jeweils von einem auszubildenden Chirurgen durchgeführt wurden, verglichen mit einem Fach‑/Oberarzt. Die dadurch höheren Kosten für die durchzuführende Prozedur erreichen dafür bis zu $2142 im Fall der Mastoidektomie [17]. Chirurgische Ausbildung braucht Zeit – ein entsprechendes Bewusstsein könnte dazu beitragen, die Zeiteinschätzung geplanter Operationen zu verbessern [2]. Die Dauer einer chirurgischen Facharztausbildung, die mehrere Jahre dauert, kann sich dadurch negativ auf die wirtschaftliche Effizienz eines Klinikbetriebs auswirken. Eine mögliche Methode zur Zeitersparnis im OP kann darin bestehen, die chirurgische Ausbildung außerhalb des OPs zu verbessern. Akademische Krankenhäuser könnten beispielsweise in Simulatortrainingsprogramme investieren. Frühere Studien haben gezeigt, dass Operationssimulationen die Leistung der AÄ im OP verbessert. AÄ, die an Simulationen teilnehmen, arbeiten tendenziell schneller als solche, die keine entsprechende Schulung erhalten [8, 17, 18]. Durch Simulationstrainings außerhalb des OPs könnten die Operationszeiten der AÄ an die der FÄ im klinischen Alltag angenähert werden, was die Operationszeiten standardisiert und die OP-Planung vereinfacht. Es soll daher hier gezeigt werden, inwiefern sich der Ausbildungsstand des durchführenden Chirurgen sowohl hinsichtlich seiner subjektiven Einschätzung von Falldauern im OP als auch hinsichtlich der benötigten Zeit zur Durchführung einer Prozedur auswirkt.

## Material und Methoden

**Figure 1:**
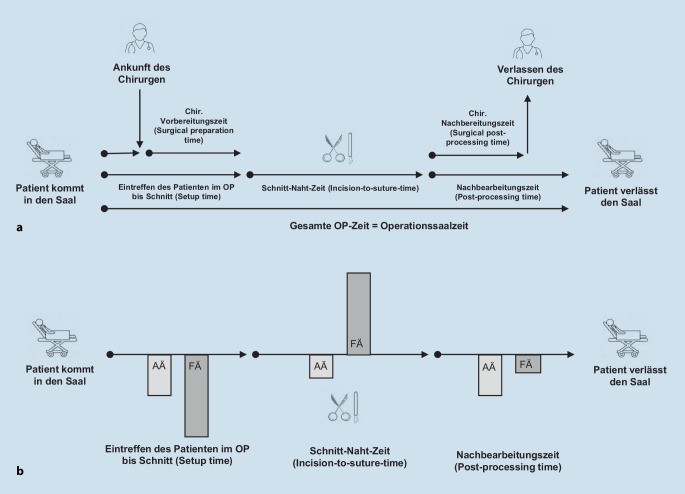

In einer retrospektiven Querschnittstudie betreffend das Jahr 2018 sind in unserer Klinik für HNO-Heilkunde, Kopf- und Halschirurgie 1809 Operationen in Intubationsnarkose analysiert worden. Eine entsprechende Darstellung erfolgt orientiert an den STROBE-Richtlinien. Die Analyse umfasste 22 verschiedene Operationsarten, die von 31 Chirurgen (12 Fachärzte (FÄ) und 19 Assistenzärzte (AÄ)) durchgeführt wurden. Zeitdauern wurden perioperativ im regulären Klinikbetrieb mithilfe von SAP NetWeaver 7.0 – Software (SAP®, Walldorf) dokumentiert. Verschiedene Zeitdauern wurden aus dieser Dokumentation extrahiert: (Abb. 1a) 1. die Zeit vom Eintreffen des Patienten im OP bis zum Eintreffen des Chirurgen; 2. die Zeit vom Eintreffen des Patienten im OP bis Schnitt („setup time“); 3. die chirurgische Vorbereitungszeit („surgical preparation time“); 4. die Schnitt-Naht-Zeit („incision-to-suture time“); 5. die Nachbearbeitungszeit („post-processing time“) und 6. die chirurgische Nachbearbeitungszeit („surgical post-processing time“) [6, 14].

Es wurden Daten zu 1873 Operationen erhoben, 64 wurden wegen der Kombination verschiedener Op.-Arten ausgeschlossen (z. B. Septumplastik plus Nasennebenhöhlen-Op. bei einem Patienten/einem Fall). Die zusätzlich anhand von Fragebögen einmalig geschätzte eigene durchschnittliche und für das Gesamtteam durchschnittliche Schnitt-Naht‑, Vorbereitungs- und Nachbereitungszeit von 10 FÄ und 17 AÄ wurden mit den objektiv erhobenen Zeiten verglichen. Der Vergleich wurde abgeschlossen für die folgenden Arten von Operationen: Halslymphknotenentfernung (*n* = 65), Mittelgesichtsrevision nach Fraktur (*n* = 39), Panendoskopie einschließlich Mikrolaryngoskopie (*n* = 378), einseitige Neck-Dissection (*n* = 13), endonasale endoskopische Nasennebenhöhlen-Op. (*n* = 213), Septumplastik (*n* = 150), Septorhinoplastik (*n* = 170), Stapesplastik (*n* = 9), Submandibulektomie (*n* = 12), Tonsillektomie (*n* = 141), Tracheotomie (*n* = 40), Tympanoplastik (*n* = 146), Tonsillektomie mit Uvulopalatopharyngoplastik (*n* = 63), intrakapsuläre Tonsillektomie (*n* = 110) und laterale Parotidektomie (*n* = 55). Insgesamt wurde der Vergleich für 1604 Operationen durchgeführt. Es ergaben sich einige Operationen (*n* = 205), die nur von FÄ durchgeführt wurden, sodass sie aus dem Vergleich ausgeschlossen werden mussten (z. B. Cochleaimplantat, Implantation Hypoglossusstimulator u. a.). Beide Vergleiche wurden mittels Mann-Whitney-U-Test auf statistische Signifikanz geprüft. Ein *p-*Wert von kleiner oder gleich 0,05 wurde als statistisch signifikant angesehen.

## Ergebnisse

**Figure 2:**
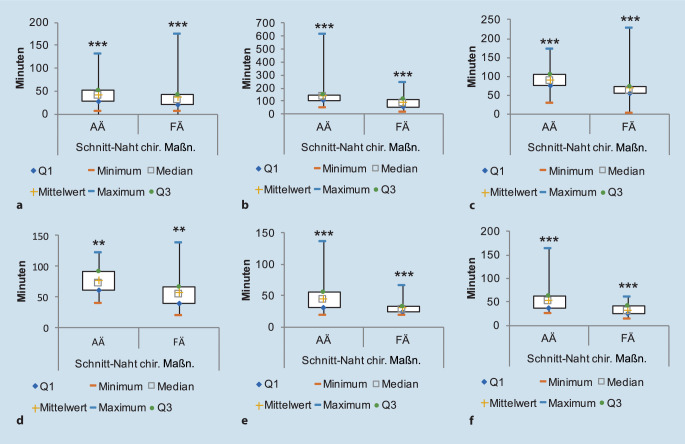

Der Vergleich der objektiven Zeiten von FÄ und AÄ zeigt signifikante Unterschiede in der Schnitt-Naht-Zeit bzw. der Summe aus Schnitt-Naht-Zeit und Zeit der chirurgischen Maßnahmen vor und nach der Op. für 6 von 15 Op.-Arten. Demnach sind FÄ bei folgenden der Op.-Prozeduren signifikant schneller verglichen mit AÄ (*p* < 0,001). Angegeben werden hier die Medianwerte: Panendoskopie einschließlich Mikrolaryngoskopie (39 min durch AÄ versus 30 min durch FÄ), endonasale endoskopische Nasennebenhöhlen-Op. (132 min durch AÄ versus 81 min durch FÄ), Septumplastik (86 min durch AÄ versus 62 min durch FÄ), Uvulopalatopharyngoplastik (71 min durch AÄ versus 54 min durch FÄ), Tonsillektomie (44 min durch AÄ versus 28 min durch FÄ), intrakapsuläre Tonsillektomie (50 min durch AÄ versus 30 min durch FÄ; Abb. 2a–f). Zusätzlich fällt bei der Auswertung auf, dass FÄ häufig länger brauchen, um überhaupt im OP zu erscheinen (Tab. 1), die durchzuführende Prozedur dann aber zügiger erledigen (Tab. 2).

**Table 1:** 

*Prozedur*	–	Eintreffen des Patienten – Eintreffen des Chirurgen	„Setup“	„Surgical preparation“	„Incision – suture“	„Post-processing“	„Surgical post-processing“
Halslymphknotenentfernung	AÄ (36)	0 (0–22)	20 (7–41)	15 (1–39)	45,5 (23–111)	11,5 (4–50)	3 (0–14)
	FÄ (29)	0 (0–15)	20 (10–53)	16 (1–36)	39 (22–79)	14,5 (4–32)	2 (0–7)
	–	*p* *=* *0,0296*	*p* = 0,6673	*p* = 0,5803	*p* = 0,0769	*p* = 0,5284	*p* *=* *0,0061*
Mittelgesichtsrevision nach Fraktur	AÄ (17)	0 (0–20)	23 (14–61)	16 (0–53)	73 (1–165)	14 (4–36)	2 (0–5)
	FÄ (22)	0 (0–12)	23 (13–61)	15 (1–36)	75 (34–255)	13,5 (7–30)	2 (0–23)
	–	*p* = 0,8970	*p* = 0,6808	*p* = 0,7558	*p* = 0,8873	*p* = 0,9773	*p* = 0,9311
Panendoskopie mit Mikrolaryngoskopie	AÄ (198)	0 (0–25)	8 (1–49)	4 (0–48)	33 (0–115)	15,5 (3–53)	2 (0–22)
	FÄ (180)	0 (0–29)	7 (1–52)	3 (0–23)	25 (5–166)	17 (3–166)	2 (0–38)
	–	*p* *=* *0,007*	*p* = 0,086	*p* *<* *0,001*	*p* *<* *0,0001*	*p* *=* *0,025*	*p* = 0,168
Nasennebenhöhlen-Op.	AÄ (46)	0 (0–19)	19 (9–39) 18 (1–48) *p* = 0,9375	14 (1–35)	115,5 (41–603)	12,5 (5–26)	2 (0–9)
	FÄ (167)	0 (0–23)		13 (0–101)	68 (9–244)	13,5 (4–56)	2 (0–29)
	–	*p* *=* *0,0182*		*p* = 0,3717	*p* *<* *0,0001*	*p* = 0,0672	*p* = 0,7524
Septumplastik	AÄ (77)	0 (0–15)	16 (4–53)	11 (0–40)	73 (28–160)	15 (5–40)	2 (0–8)
	FÄ (73)	0 (0–13)	14 (4–36)	9 (0–24)	53 (7–203)	14 (3–29)	2 (0–11)
	–	*p* *=* *0,0326*	*p* = 0,0871	*p* = 0,0883	*p* *<* *0,0001*	*p* = 0,1837	*p* = 0,9508
Submandibulektomie	AÄ (10)	0 (0–12)	20,5 (14–32)	18 (3–26)	76 (45–110)	13 (8–20)	2,5 (1–6)
	FÄ (2)	5 (5–5)	23 (19–27)	12,5 (12–13)	57,5 (43–72)	9,5 (9–10)	3 (3–3)
	–	*p* = 0,1587	*p* = 1,0000	*p* = 0,3877	*p* = 0,2374	*p* = 0,2785	*p* = 0,9128
Tonsillektomie	AÄ (92)	0 (0–12)	8 (2–28)	4 (0–16)	35 (14–122)	15 (1–32)	2 (0–9)
	FÄ (49)	0 (0–12)	7 (1–20)	3 (0–24)	23 (1–59)	15 (4–34)	2 (0–19)
	–	*p* = 0,1148	*p* = 0,4233	*p* = 0,8034	*p* *<* *0,0001*	*p* = 0,9544	*p* = 0,6217
Tracheotomie	AÄ (27)	0 (0–13)	28 (10–56)	15,5 (2–31)	52 (6–96)	16,5 (5–58)	3,5 (0–10)
	FÄ (13)	0 (0–3)	19 (13–28)	19 (13–28)	45 (26–78)	17 (13–22)	3 (2–4)
	–	*p* = 0,6979	*p* = 0,7362	*p* = 0,1680	*p* = 0,5345	*p* = 0,8551	*p* = 0,7028
Uvulopalatopharyngoplastik	AÄ (42)	0 (0–10)	7,5 (3–21)	4 (0–12)	66 (35–112)	17 (0–55)	2 (0–9)
	FÄ (21)	0 (0–16)	9 (4–19)	3 (1–11)	49 (14–130)	13 (5–44)	2 (0–4)
	–	*p* *=* *0,0094*	*p* *=* *0,0068*	*p* = 0,3687	p = 0,0018	*p* = 0,2061	p = 0,0154
Laterale Parotidektomie	AÄ (6)	0 (0–0)	34 (18–39)	32 (13–34)	121 (58–176)	8 (5–13)	2 (1–8)
	FÄ (49)	0 (0–39)	26,5 (5–54)	23 (1–44)	124 (22–280)	16 (7–55)	4 (0–11)
	–	*p* = 0,3080	*p* = 0,4817	*p* = 0,1675	*p* = 0,6560	p = 0,0016	*p* = 0,1765

Stichprobengrößen sind in Klammern angegeben

Ein *p*-Wert kleiner oder gleich 0,05 wurde als statistisch signifikant angesehen (*kursiv*)

**Table 2:** 

*Prozedur*	–	Gesamte Op.-Dauer	„Setup + post-processing“	„Surgical preparation + surgical post-processing“	„Surgical preparation + incision – suture + surgical post-processing“
Halslymphknotenentfernung	AÄ (36)	83,5 (43–165)	32 (17–84)	17 (3–42)	66,5 (36–130)
	FÄ (29)	75 (45–120)	36 (21–63)	17 (2–31)	58 (33–110)
	–	*p* = 0,1446	*p* = 0,4717	*p* = 0,7771	*p* = 0,1102
Mittelgesichtsrevision nach Fraktur	AÄ (17)	121 (32–215)	42 (29–75)	17,5 (2–57)	87 (11–192)
	FÄ (22)	109 (57–341)	38,5 (21–86)	17 (2–45)	83 (50–300)
	–	*p* = 0,9435	*p* = 0,7020	*p* = 0,8243	*p* = 0,7988
Panendoskopie mit Mikrolaryngoskopie	AÄ (198)	59 (8–176)	25 (6–97)	6 (0–53)	39 (7–132)
	FÄ (180)	52,5 (24–216)	26 (8–77)	5 (0–40)	30 (6–175)
	–	*p* *=* *0,0013*	*p* = 0,1514	*p* *=* *0,0116*	*p* *<* *0,0001*
Nasennebenhöhlen-Op.	AÄ (46)	148 (68–629)	31 (20–54)	16 (2–43)	132,5 (48–613)
	FÄ (167)	98,5 (31–253)	34 (12–70)	15 (0–104)	81,5 (12–235)
	–	*p* *<* *0,0001*	*p* = 0,0688	*p* = 0,6013	*p* *<* *0,0001*
Septumplastik	AÄ (77)	107 (64–202)	32 (12–67)	13 (2–42)	86 (30–171)
	FÄ (73)	80,5 (19–244)	27 (13–52)	11 (1–29)	62,5 (4–229)
	–	*p* *<* *0,0001*	*p* *=* *0,0109*	*p* = 0,1604	*p* *<* *0,0001*
Submandibulektomie	AÄ (10)	104,5 (89–150)	33,5 (26–50)	21 (4–30)	87,5 (72–135)
	FÄ (2)	90 (71–109)	32,5 (28–37)	15,5 (15–16)	73 (59–87)
	–	*p* = 0,5912	*p* = 0,8266	*p* = 0,4513	*p* = 0,2819
Tonsillektomie	AÄ (92)	62 (37–165)	22,5 (8–45)	5 (0–21)	44 (19–137)
	FÄ (49)	46 (7–85)	23 (6–49)	5 (0–43)	28 (19–67)
	–	*p* *<* *0,0001*	*p* = 0,9806	*p* = 0,9197	*p* *<* *0,0001*
Tracheotomie	AÄ (27)	78,5 (6–179)	14 (0–114)	18 (6–38)	67,5 (12–127)
	FÄ (13)	45 (26–109)	0 (0–45)	22 (15–32)	55 (32–95)
	–	*p* = 0,2332	*p* = 0,2173	*p* = 0,7362	*p* = 0,2573
Uvulopalatopharyngoplastik	AÄ (42)	92 (63–157)	25 (4–63)	6 (2–15)	71,5 (40–122)
	FÄ (21)	68 (32–180)	24 (13–60)	5 (2–11)	54 (19–139)
	–	*p* *=* *0,004*	*p* = 0,8609	*p* *=* *0,0399*	*p* *=* *0,0010*
Laterale Parotidektomie	AÄ (6)	164,5 (84–219)	41 (26–48)	32 (16–42)	158 (74–211)
	FÄ (49)	162 (68–328)	44 (16–78)	28 (3–50)	146 (53–308)
	–	*p* = 0,818	*p* = 0,2240	*p* = 0,6229	*p* = 0,6365

Ein *p*-Wert kleiner oder gleich 0,05 wurde als statistisch signifikant angesehen (*kursiv*)

Darüber hinaus unterscheiden sich FÄ und AÄ wesentlich in ihrer subjektiven Einschätzung hinsichtlich geschätzter Zeitdauern im OP (Abb. 1b, Tab. 3). Die durch FÄ geschätzte Nachbereitungszeit wich bei 2 von 22 Op.-Arten von der objektiven Zeit ab (jeweils *p* < 0,05), die durch AÄ geschätzte Zeit bei 7 von 15 Op.-Arten (jeweils *p* < 0,05). Hinsichtlich der Schnitt-Naht-Zeit verschätzten sich FÄ bei 7 von 22 (jeweils *p* < 0,05), AÄ bei 3 von 15 (jeweils *p* < 0,05) Op.-Arten. Die durch FÄ geschätzte Vorbereitungszeit wich bei 16 von 22 Op.-Arten signifikant von der objektiven Zeit ab (jeweils *p* < 0,05), bei AÄ bei 7 von 15 (jeweils *p* < 0,001). Vor- und Nachbereitungszeiten wurden durch FÄ unter‑, Schnitt-Naht-Zeiten überschätzt. AÄ unterschätzten alle Zeiten (Abb. 1b).

**Table 3:** 

*Prozedur*	Fachärzte		Assistenzärzte	
Cochleaimplantat	Obj. (92)	56,5 (12–107)	n.a.	
	Sub. (8)	20,0 (15–30)		
	–	*p* *<* *0,0001*		
Halslymphknotenentfernung	Obj. (28)	35,0 (11–89)	Obj. (34)	34,5 (10–80)
	Sub. (9)	20,0 (19–30)	Sub. (14)	10,0 (5–25)
	–	*p* *=* *0,0002*	–	*p* *<* *0,0001*
Implantation eines Hypoglossusstimulators	Obj. (31)	66,0 (4–123)	n.a.	
	Sub. (6)	30,0 (15–60)		
	–	*p* *=* *0,0094*		
Mittelgesichtsrevision nach Fraktur	Obj. (44)	37 (15–97)	Obj. (16)	39,5 (15–114)
	Sub. (8)	17,5 (10–30)	Sub. (14)	15 (5–20)
	–	*p* *=* *0,0006*	–	*p* *<* *0,0001*
Panendoskopie mit Mikrolaryngoskopie	Obj. (177)	10 (1–58)	Obj. (191)	12 (2–97)
	Sub. (9)	10 (5–20)	Sub. (16)	10 (5–45)
	–	*p* = 0,8863	–	*p* = 0,2095
Mastoidektomie	Obj. (13)	40 (9–96)	n.a.	
	Sub. (8)	15 (15–25)		
	–	*p* *=* *0,0448*		
Einseitige Neck-Dissection	Obj. (11)	49 (20–102)	n.a.	
	Sub. (9)	20 (15–30)		
	–	*p* *=* *0,0005*		
Nasennebenhöhlen-Op.	Obj. (162)	31 (2–111)	Obj. (46)	31,5 (14–73)
	Sub. (9)	15 (15–25)	Sub. (15)	15 (5–20)
	–	*p* *=* *0,0002*	–	*p* *<* *0,0001*
Septumplastik	Obj. (72)	24 (5–59)	Obj. (75)	26 (6–86)
	Sub. (10)	15 (10–20)	Sub. (15)	15 (5–20)
	–	*p* *=* *0,0092*	–	*p* *<* *0,0001*
Septorhinoplastik	Obj. (165)	36 (7–103)	n.a.	
	Sub. (9)	15 (10–25)		
	–	*p* *<* *0,0001*		
Sialendoskopie	Obj. (16)	27 (14–38)	n.a.	
	Sub. (9)	15 (10–30)		
	–	*p* *=* *0,0355*		
Stapesplastik	Obj. (8)	50 (23–87)	n.a.	
	Sub. (7)	20 (15–20)		
	–	*p* *=* *0,0013*		
Submandibulektomie	Obj. (2)	35,5 (32–39)	Obj. (10)	38 (17–56)
	Sub. (9)	20 (5–30)	Sub. (13)	15 (5–25)
	–	*p* *=* *0,0398*	–	*p* *=* *0,0001*
Tracheotomie	Obj. (3)	48 (36–56)	Obj. (12)	48 (17–72)
	Sub. (9)	15 (10–20)	Sub. (17)	15 (6–60)
	–	*p* *=* *0,0101*	–	*p* *<* *0,0001*
Tympanoplastik	Obj. (140)	38 (7–102)	n.a.	
	Sub. (8)	17,5 (10–20)		
	–	*p* *<* *0,0001*		
Laterale Parotidektomie	Obj. (48)	49,5 (8–90)	Obj. (6)	66 (31–73)
	Sub. (8)	22,5 (15–30)	Sub. (14)	15 (5–30)
	–	*p* *=* *0,0001*	–	*p* *=* *0,0006*
Totale Parotidektomie	Obj. (10)	66 (43–175)	n.a.	
	Sub. (7)	25,0 (20–35)		
	–	*p* *=* *0,0007*		

Ein *p*-Wert kleiner oder gleich 0,05 wurde als statistisch signifikant angesehen (*kursiv*)

*n.a.* keine Daten verfügbar

## Diskussion

Ein Ziel unserer Studie war es festzustellen, ob überhaupt und welche Arten von Betriebszeiten verlängert werden, wenn Operationen von AÄ durchgeführt werden. Es ergab sich für fast alle untersuchten Prozeduren eine signifikant längere erforderliche Zeit zur Durchführung, wenn AÄ operierten (Tab. 1 und 2). Nur für drei Arten von Operationen (Mittelgesichtsrevision bei Fraktur, Submandibulektomie und Tracheotomie) konnte kein signifikanter Unterschied gefunden werden, möglicherweise wegen der z. T. geringen Stichprobengröße. Darüber hinaus sollte eine Aussage zu möglichen Fehlerquellen bei der OP-Planung anhand objektiver Zeiteinschätzungen durch Chirurgen gemacht werden. Es ergab sich dabei, dass FÄ insbesondere Fehleinschätzungen hinsichtlich der Vor- und Nachbereitungszeiten chirurgischer Prozeduren machen, nämlich bei 7 von 15 Arten von Operationen. Auch die Schnitt-Naht-Zeit wird bei 3 von 15 Op.-Arten falsch eingeschätzt. Noch schlechter schätzen AÄ die erforderlichen Zeiten im OP ein – sie neigen dabei dazu, die erforderlichen Zeiten noch mehr zu unterschätzen als FÄ.

In unserer Studie wurden die Operationsdauern an unserem Klinikum berücksichtigt. Andere medizinische Zentren verwenden möglicherweise andere Operationstechniken oder haben eine andere personelle Besetzung im ärztlichen Bereich. Dies kann zu unterschiedlichen objektiven Zeitdauern der durchführenden Chirurgen führen. Darüber hinaus kann es je nach Organisation des OPs zu unterschiedlichen Vor- und Nachbearbeitungszeiten kommen (z. B. bei mehr oder weniger Personal im anästhesiologischen oder pflegenden Bereich oder anderer Ausstattung hinsichtlich Vorbereitungsräumen und Aufwachräumen). Darüber hinaus können unterschiedliche Organisationsformen zu einem höheren Patientenstrom führen, z. B. durch überlappende Narkoseeinleitungen. Letztlich sind aber nach unseren Erfahrungen und nach dem Austausch mit Mitarbeitern anderer HNO-Abteilungen die Hürden im Alltag ähnlich, sodass unsere Ergebnisse a. e. auch andernorts anwendbar sein sollen.

Unsere Studie hat gezeigt, dass die Aufgaben des Chirurgen, der die Operation durchführt, bei der OP-Planung berücksichtigt werden müssen. FÄ betreten beispielsweise den OP manchmal verspätet, führen die Operation dann aber schneller durch als AÄ. Ein möglicher Grund für den verspäteten Eintritt der FÄ in den OP ist, dass sie oft mehrere Aufgaben haben, wie zum Beispiel die Leitung einer Bettenstation sowie zusätzliche Sprechstunden. Frühere Studien haben gezeigt, dass die Abwesenheit von Chirurgen ein häufiger Grund für Verzögerungen beim Operationsbeginn ist [12]. Daher sollte die OP-Planung die anderen Pflichten des vorgesehenen Chirurgen, wie Besprechungen und Sprechstunden, berücksichtigen. Darüber hinaus scheinen mehr Kommunikation durch und an den Chirurgen bei unerwarteter Verspätung und Erinnerungen an den Chirurgen Strategien zu sein, die helfen könnten, Verzögerungen zu vermeiden [7, 16].

Die Unterschiede in den geschätzten OP-Zeiten zwischen AÄ und FÄ lassen sich durch ihren unterschiedlichen Erfahrungsschatz erklären. AÄ scheinen „optimistischer“ zu sein, was sich in ihren (unter)geschätzten Schnitt-Naht-Zeiten, Nachbearbeitungs- und Vorbereitungszeiten zeigt. Lediglich im Hinblick auf ihre individuelle Operationsdauer erwarteten FÄ kürzere Zeiträume als die AÄ. Bei 6 von 15 Operationsarten rechneten FÄ mit einer geringeren Operationszeit. Bei 4 dieser 6 Operationsarten waren die tatsächlichen OP-Zeiten kürzer als die der AÄ. Diese vier Operationen (Septumplastik, Tonsillektomie, Tonsillektomie mit Uvulopalatopharyngoplastik und intrakapsuläre Tonsillektomie) werden typischerweise für den Unterricht verwendet und werden oft von AÄ durchgeführt. Daher kann davon ausgegangen werden, dass sich FÄ bewusst sind, dass AÄ für diese Verfahren mehr Zeit benötigen. Dieses Wissen garantiert jedoch nicht, dass dies bei der OP-Planung immer berücksichtigt wird. Es kann auch eine Verhaltensverzerrung seitens des planenden – häufig hierarchisch höher gestellten – Chirurgen vorliegen.

In unserer Studie wurden die Zeitschätzungen für unterschiedliche Op.-Prozeduren einmalig kumulativ anhand eines Fragebogens abgefragt, nicht vor jedem Eingriff separat. Eine spezifischere, fallbezogene Datenerhebung etwa durch die Dokumentation geschätzter Zeitdauern im Rahmen des präoperativen Team-Timeouts würde eine noch präzisere spezifischere Einschätzung hinsichtlich Prozedur und zugehörigem Operateur*in ermöglichen. Andererseits bleibt fragwürdig, ob eine derartig spezifische Korrelationen, die an Verknüpfungen eines bestimmten Falls mit einem bestimmten Operateur*in gebunden sind, für eine tägliche OP-Planung, die z. B. aufgrund von unvorhersehbaren Abwesenheiten auch etwas Flexibilität anbieten muss, überhaupt zielführend wären.

Unsere Ergebnisse deuten darauf hin, dass die Einschätzungen von AÄ und FÄ in Bezug auf die Zeit von der Inzision bis zur Naht einigermaßen genau sind, aber beide unterschätzen häufig die notwendigen Ressourcen (d. h. Zeit und Personal) bei der Bewertung der Vorbereitungszeiten vor dem Schneiden sowie der Nachbearbeitung (Abb. 1b). In unserer Studie waren die tatsächlichen Vorbereitungszeiten vor der Inzision fast dreimal so lang wie die geschätzten Zeiten (Tab. 1, Abb. 1b). Auch hinsichtlich der erforderlichen Nachbereitungszeiten treten Fehleinschätzungen auf, die zu falschen Planungen führen können. Dadurch kommt es im Klinikalltag nicht selten zu der Beobachtung, dass es zu einem Stau in der Abarbeitung von Op.-Punkten kommt, weil Nachbereitungszeiten unterschätzt wurden und das „überlappende“ Einschleusen von Patient*innen in den OP vonseiten der anästhesiologischen Kollegen z. B. wegen Personalknappheit nicht möglich ist. Aber wie sollen Chirurgen auch ein Gefühl dafür haben, wie lange die Vor- und Nachbereitungszeiten einer Operation sind? Sie betreten den OP zum Operieren und verlassen ihn nicht selten anschließend recht zügig, häufig wegen des Aufkommens zusätzlicher Aufgaben außerhalb des OPs. Wenn Chirurgen an einen chirurgischen Eingriff denken, denken sie hauptsächlich an den Prozess, an dem sie teilnehmen, und verlieren die Bedürfnisse vor und nach der Operation aus den Augen. Dieser „Planungsfehler“ wurde erstmals 1979 von Kahneman und Tversky beschrieben. Es handelt sich um ein Phänomen, bei dem Vorhersagen über die Zeit, die für eine Aufgabe benötigt wird, einen „Optimismus-Bias“ aufweisen und zu einer Unterschätzung des Zeitbedarfs führen. Dies kann unabhängig vom Wissen des Einzelnen geschehen, dass frühere Aufgaben ähnlicher Art länger gedauert haben als allgemein geplant. Kahneman und Tversky erklärten den Trugschluss damit, dass Planer sich eher auf das optimistischste Szenario für die Erledigung einer Aufgabe konzentrieren, anstatt anhand ihrer bisherigen Erfahrungen zu beurteilen, wie viel Zeit eine ähnliche Aufgabe tatsächlich benötigt [13]. Mit unseren Daten zeigen wir erstmals, dass einige chirurgische Eingriffe in der HNO leichter abzuschätzen sind als andere, z. B. sind chirurgische Falldauern einfacher abzuschätzen, wenn es sich um eine oft unkomplizierte Prozedur wie eine Tonsillektomie oder eine Septumplastik handelt. Dies entspricht den Erkenntnissen von Gordon et al., die feststellten, dass Laparoskopien an den Johns Hopkins Medical Institutions um 42 % von der geschätzten Zeit abwichen, während Hysterektomien, Leistenhernien-Operationen und Prostatektomien nur bis zu 4 % variierten [9]. Unsere Daten stützen die Behauptung, dass einige Arten von Operationen von Natur aus schwerer vorhersehbar sind als andere.

Wir haben jedoch fast 2000 Verfahren analysiert, was es uns ermöglichte, die durchschnittliche Dauer bestimmter Verfahren zu erheben. Trotz unvorhersehbarer Ereignisse, wie Managementproblemen, Patiententransport in den OP oder Komplikationen/Schwierigkeiten, die während einer Operation auftreten können, muss die Verwaltung und Planung des OPs auf objektiven Informationen basieren [3, 19]. Die Dauer einer Operation hängt von den individuellen Eigenschaften des Patienten, dem Können des Chirurgen und der Routine ab, mit der der Eingriff durchgeführt wird. Dies lässt sich an den zeitlichen Unterschieden für einen Eingriff eines HNO-Assistenzarztes im Vergleich zu einem Facharzt zeigen (Abb. 2). Dies ist ein wichtiges Ergebnis, da OP-Manager in der Lage sein könnten, bessere Fallpläne zu erstellen, indem sie Schätzungen von Chirurgen und die objektiven Zeiten in verschiedene Teile des Op.-Prozesses einbeziehen. Die Erkenntnis, dass es keine „falschen“ OP-Zeiten, sondern nur „Wahrscheinlichkeiten“ gibt, ist für ein Op.-Team und letztlich für die Organisation des gesamten Krankenhauses von großem Wert. In einem so komplexen System wie einer chirurgischen Lehrklinik sind letztlich nicht alle Einflussfaktoren messbar. In der Praxis sind Fälle, die hinsichtlich ihrer zeitlichen Durchführbarkeit einfach beurteilt werden können, an einem Universitätsklinikum äußerst selten, wie beispielsweise ein ansonsten völlig gesunder Patient zur Tonsillektomie, die von einem Facharzt durchgeführt wird. Es gibt viel mehr Patienten an Universitätskliniken, für die zusätzliche Verzögerungsmaßnahmen ergriffen werden müssen, beispielsweise aufgrund von verkomplizierenden Vorerkrankungen. All diese Maßnahmen brauchen Zeit.

Ähnlich wie in unserer Studie haben Vinden et al. festgestellt, dass ein breites Spektrum chirurgischer Eingriffe in der Lehre deutlich mehr Zeit in Anspruch nimmt als in nichtlehrenden Krankenhäusern. Sie postulierten, dass die Größenordnung dieses Unterschieds groß genug ist, um potenziell direkte und indirekte Kosten zu verursachen sowie die Effizienz der Einrichtung zu beeinträchtigen [20].

Ein Op.-Team ist eine hierarchisch gegliedertes Konstrukt von Menschen unterschiedlicher Berufsgruppen, das anfällig für Störungen ist. Darüber hinaus bieten unterschiedliche Individualzielsetzungen der verschiedenen Teammitglieder, die unter wirtschaftlichem Leistungsdruck stehen, potenzielle Konfliktquellen im Arbeitsalltag. Dies kann sich negativ auf die Patientensicherheit und die Effizienz des Krankenhausmanagements auswirken. In dieser Situation ist ein wichtiges Instrument zur Teambildung im OP nach unserer Beobachtung ein „Team-Timeout“, das zu Beginn jedes chirurgischen Eingriffs durchgeführt wird. Das Sammeln prospektiver Daten von Zeitschätzungen durch Chirurgen während das Team-Timeouts wäre eine sinnvolle prospektive Erweiterung unserer Datenerhebung.

Zusammenfassend lässt sich sagen, dass es viele potenzielle Fehlerquellen bei der OP-Planung gibt, die beachtet werden müssen, um eine wirtschaftliche Rationalität zu ermöglichen.

## Fazit für die Praxis

- Für die OP-Zeiteneinschätzung muss die Erfahrung des durchführenden Chirurgen berücksichtigt werden. Durch AÄ durchgeführte Lehreingriffe benötigen mehr Zeit, was bei der täglichen Operationsplanung nicht selten vernachlässigt wird.
- Eine verbesserte Planung durch verminderte subjektive Fehleinschätzung kann möglicherweise mithilfe spezieller Algorithmen gelingen.
- Möglicherweise ist es von sinnvoll, Vor- und Nachbereitungszeiten gemeinsam mit dem Pflegepersonal und dem Anästhesisten zu bestimmen und in der OP-Planung zu berücksichtigen.
